# Hemisynapse Formation Between Target Astrocytes and Cortical Neuron Axons *in vitro*

**DOI:** 10.3389/fnmol.2022.829506

**Published:** 2022-03-21

**Authors:** Zenghui Teng, Kurt Gottmann

**Affiliations:** Institute of Neuro- and Sensory Physiology, Medical Faculty, Heinrich Heine University Düsseldorf, Düsseldorf, Germany

**Keywords:** target astrocytes, synapse formation, hemisynapse, synaptogenic protein, LRRTM2

## Abstract

One of the most fundamental organizing principles in the mammalian brain is that neurons do not establish synapses with the other major cell type, the astrocytes. However, induced synapse formation between neurons and astrocytes appears conceivable, because astrocytes are well known to express functional ionotropic glutamate receptors. Here, we attempted to trigger synapse formation between co-cultured neurons and astrocytes by overexpressing the strongly synaptogenic adhesion protein LRRTM2 in astrocytes physically contacted by cortical axons. Interestingly, control experiments with immature cortical astrocytes without any overexpression resulted in the induction of synaptic vesicle clustering in contacting axons (hemisynapse formation). This synaptogenic activity correlated with the endogenous expression of the synaptogenic protein Neuroligin1. Hemisynapse formation was further enhanced upon overexpression of LRRTM2 in cortical astrocytes. In contrast, cerebellar astrocytes required overexpression of LRRTM2 for induction of synaptic vesicle clustering in contacting axons. We further addressed, whether hemisynapse formation was accompanied by the appearance of fully functional glutamatergic synapses. We therefore attempted to record AMPA receptor-mediated miniature excitatory postsynaptic currents (mEPSCs) in innervated astrocytes using the whole-cell patch-clamp technique. Despite the endogenous expression of the AMPA receptor subunits GluA2 and to a lesser extent GluA1, we did not reliably observe spontaneous AMPA mEPSCs. In conclusion, overexpression of the synaptogenic protein LRRTM2 induced hemisynapse formation between co-cultured neurons and astrocytes. However, the formation of fully functional synapses appeared to require additional factors critical for nano-alignment of presynaptic vesicles and postsynaptic receptors.

## Introduction

Synapse formation is an essential neurodevelopmental process that enables neurons to transfer, process, and store information in highly connected cellular networks. Molecular interactions between pre- and postsynaptic partner neurons are thought to be needed to establish exactly aligned pre- and postsynaptic compartments (Südhof, 2018; Sanes and Zipursky, 2020; Südhof, 2021). A relatively simple, but fundamental molecular mechanism might be the transsynaptic interaction of pre- and postsynaptically localized transmembrane receptor proteins (Südhof, 2008; Shen and Scheiffele, 2010; Siddiqui and Craig, 2011). These transsynaptic adhesion molecules would then initiate pre- and postsynaptic signaling pathways ultimately resulting in both presynapse formation and postsynaptic differentiation (Takahashi and Craig, 2013; Südhof, 2017; Gomez et al., 2021).

A number of transsynaptic adhesion molecules that induce synaptic vesicle clustering in “presynaptic” neurons have been identified and have been termed synaptogenic proteins (Shen and Scheiffele, 2010; Siddiqui and Craig, 2011; de Wit and Ghosh, 2014; Südhof, 2017). In a landmark study, Scheiffele et al. (2000) demonstrated that expression of the postsynaptic adhesion protein Neuroligin1 in non-neural HEK293 cells induced synaptic vesicle clustering in contacting axons reminiscent of presynapse formation. Other neuronal adhesion proteins such as e.g., SynCAM1 (Biederer et al., 2002), NGL-3 (Woo et al., 2009), Slitrks (Yim et al., 2013), and LRRTM2 (de Wit et al., 2009; Ko et al., 2009; Linhoff et al., 2009) were subsequently described to exhibit a similar synaptogenic activity with LRRTM2 showing a particularly strong vesicle clustering effect. These induced vesicle clusters were capable of releasing vesicles resulting in postsynaptic currents when glutamate receptors were highly overexpressed in non-neural cells (Biederer et al., 2002; Fu et al., 2003; Sara et al., 2005; de Wit et al., 2009; Linhoff et al., 2009). However, this type of forced presynaptic differentiation was not accompanied by the formation of an organized postsynaptic specialization, and therefore the term hemisynapse formation was coined (Craig et al., 2006) to describe this partial synapse formation. Hemisynapse formation does not only occur with postsynaptic adhesion proteins acting on contacting neurons, but also with presynaptic adhesion proteins acting on neurons that serve as postsynaptic targets (Siddiqui and Craig, 2011; Südhof, 2017). As an example, expression of beta-neurexin in fibroblasts induced postsynaptic differentiation in neurons contacted by these fibroblasts (Graf et al., 2004; Craig and Kang, 2007).

Overexpression of these synaptogenic proteins in neurons contacting other neurons in standard neuronal cell cultures led to the induction of additional *bona fide* synapses consisting of both pre- and postsynaptic specializations. This has been demonstrated for prototypical synaptogenic proteins such as Neuroligin1, SynCAM1, and LRRTM2 (Chih et al., 2005; Sara et al., 2005; de Wit et al., 2009; Ko et al., 2009; Dagar and Gottmann, 2019). In addition, these induced synapses were fully functional as demonstrated by patch-clamp recordings of miniature excitatory postsynaptic currents (mEPSCs) that were mediated by AMPA receptors (Sara et al., 2005; Wittenmayer et al., 2009; Stan et al., 2010). From these observations, it appears conceivable that synaptogenic proteins might also be able to induce *bona fide* functional synapses if expressed in the second major neural cell type, the astrocytes. Cortical and cerebellar astrocytes are well known to endogenously express functional AMPA receptors (e.g., Sontheimer et al., 1988; von Blankenfeld and Kettenmann, 1991; Burnashev et al., 1992; Gallo et al., 1994; David et al., 1996; Ceprian and Fulton, 2019), but do not form synapses with neurons *in vivo*. In line with a potential of astrocytes to form artificial synapses with neurons, non-standard glial cell types, i.e., NG2 cells (Bergles et al., 2010; Sakry et al., 2011) and some glioblastoma cells (Venkataramani et al., 2019; Venkatesh et al., 2019) have been described to establish functional, AMPA receptor containing synapses with presynaptic neurons. However, whether artificial *bona fide* synapse formation is induced by expression of synaptogenic proteins in cultured astrocytes, which are in physical contact with neuronal axons, has not been investigated.

In this paper, we used expression of the postsynaptic adhesion protein LRRTM2 in cultured cortical and cerebellar astrocytes that were contacted by cortical axons to study whether artificial formation of fully functional synapses can occur in neuron-astrocyte pairs. Unexpectedly, we found that immature cortical astrocytes induced synaptic vesicle clusters in contacting axons even without LRRTM2 expression, while cerebellar astrocytes required LRRTM2 expression for induction of synaptic vesicle clustering. This finding correlated with the expression of Neuroligin1 in cortical astrocytes. Because both types of cultured astrocytes expressed AMPA receptor subunits, we expected to observe functional synapses in LRRTM2 expressing astrocytes. However, patch-clamp recordings in LRRTM2 expressing astrocytes contacted by axons did not reliably reveal AMPA receptor-mediated mEPSCs This indicates that although hemisynapse formation is induced by a single type of synaptogenic protein, artificial formation of fully functional synapses in neuron-astrocyte pairs requires additional molecular mechanisms.

## Materials and Methods

All experiments were done in accordance with the relevant guidelines.

### Cell Culture

Co-cultures of cortical explants and dissociated astrocytes or dissociated cortical neurons (positive controls) were done as described previously (Gottmann et al., 1997; Mohrmann et al., 1999). In brief, cortical and cerebellar astrocytes were obtained from newborn (P0) mouse cortex and P7 mouse cerebellum (brains from C57BL/6 wildtype mice) by mechanical dissociation after trypsin treatment, and further cultivation for 10–12 days in flasks with BME medium (Gibco) containing 10% FBS, L-glutamine (2 mM), glucose (20 mM), insulin transferrin selenium A (ITS) and penicilline-streptomycine (1%). This pre-cultivation step of dissociated cells was done to increase the number of astrocytes and to obtain cultures dominated by proliferating astrocytes. In cerebellar cultures, this might have led to a relative loss of Bergmann glial cells as indicated by the high percentage of GluA2 expressing astrocytes (see section “Results”). As positive controls, dissociated cortical neurons were prepared by dissociating the cortical tissue from cortices of E18-19 embryos (C57BL/6 wildtype mice) after trypsin treatment.

**FIGURE 1:**
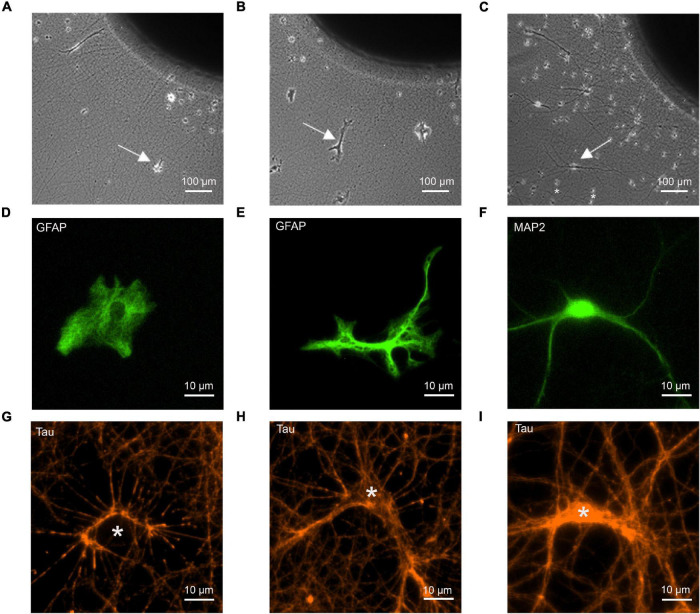
Co-cultures of cortical explants with axon outgrowth and immature astrocytes as target cells. The following target cells were added to explants: **(A,D,G)** cortical astrocytes; **(B,E,H)** cerebellar astrocytes; **(C,F,I)** cortical neurons (control). **(A–C)** Photomicrographs of co-cultures of cortical explants with axon outgrowth and target cells (white arrows indicate target cells). 12 days of co-culture for target cells. Explants were cultured 5 days before addition of dissociated target cells. In C small cells (marked by *) that occasionally migrated out of the explant (presumably microglia cells) are present in addition to co-cultured neurons. These cells were clearly distinguished from neurons and astrocytes by their different morphology. **(D–F)** Immunostaining of target cells for GFAP **(D,E)** and MAP2 **(F)** to visualize the morphology of target cells. **(G–I)** Immunostaining of outgrowing axons for tau to demonstrate formation of physical contacts between axons and target cells. White stars indicate the cell bodies of target cells. Cells in **(D–I)** are not identical.

Cortical explants were prepared by cutting the occipital cortex from cortices of E18-19 embryos (C57BL/6 wildtype mice) into tissue blocks of 0.5–0.8 mm diameter. These explants were placed in the center of polyornithine-coated glass coverslips, and cultured for 5 days with Neurobasal (NB) medium (Gibco) containing 2% B27 supplement, 0.5% Glutamax-1 supplement (Gibco) and 1% penicilline-streptomycine. For co-cultures, re-dissociated astrocytes or freshly dissociated neurons were added to the explant cultures and were further co-cultured for 12 days (see Figure 1).

### Magnetofection and Plasmids

Magnetic nanoparticles (NeuroMag; OZ Biosciences) were used to transfect the co-cultured astrocytes using a previously described transfection procedure (van Stegen et al., 2017). In brief, mixed complexes including plasmid DNA and magnetic nanoparticles in NB medium without supplements were incubated for 20 min at room temperature. After that, the DNA/NeuroMag-complexes were added to the cultures in 6-well plates for 30 min at 37°C. An oscillating magnetic plate (Magnetofection™, magnefect LT; nanoTherics) was used to enhance the transfection efficiency. Fresh NB medium was added after transfection. The plasmids pEGFP-N1 (Clontech) and myc-LRRTM2 (gift from Dr. J. de Wit, Leuven, Belgium) were used. Co-expression of myc-LRRTM2 and EGFP in astrocyte co-transfection experiments was confirmed by immunocytochemical staining for the myc tag (Supplementary Figure 1).

### Immunocytochemistry

Immunocytochemistry was performed using a standard staining protocol as described previously (Nieweg et al., 2015). The following primary antibodies were used: Anti-vGlut1 (Guinea pig polyclonal, 1:1,000, Synaptic Systems Cat# 135304, RRID: AB_887878, Göttingen, Germany); Anti-GFAP (Chicken polyclonal, 1:1,000, Millipore Cat# AB5541, RRID: AB_177521, Burlington, MA), United States; Anti-MAP2 (Chicken polyclonal, 1:1,000, Abcam Cat# Ab92434, RRID: AB_2138147, Cambridge, United Kingdom); Anti-Tau (Monoclonal mouse, 1:1,000, Synaptic Systems Cat# 314011, RRID: AB_10805762, Göttingen, Germany). Anti-NLG1 (Rabbit polyclonal, 1:1,000, Synaptic Systems Cat# 129003, RRID: AB_887746, Göttingen, Germany); Anti-GluA1 (Rabbit polyclonal, 1:1,000, Sigma-Aldrich Cat# AB 1504, RRID: AB_2113602, St. Louis, MO, United States); Anti-GluA2 (Guinea pig polyclonal, 1:500, Synaptic Systems Cat# 182105, RRID: AB_2619875, Göttingen, Germany). Anti-myc (Monoclonal mouse, 1:1,000, Thermo Fisher Cat#_46-0603, RRID: AB_2556560, MA, United States). The GluA1 and GluA2 antibodies were validated in this study by staining standard cultures of mouse cortical neurons.

The secondary antibodies used were: AF555 goat anti-mouse (1:1,000, Cat# A-21424, RRID: AB_1417801), AF555 goat anti-pig (1:1,000, Cat# A-21435, RRID: AB_2535856), AF555 goat anti-rabbit (1:1,000, Cat# A-21429, RRID: AB_2535850), AF555 goat anti-chicken (1:1,000, Cat# A-21437, RRID: AB_2535858); AF488 goat anti-mouse (1:1,000, Cat# A-11029, RRID: AB_2534088), AF488 goat anti-guinea pig (1:1,000, Cat# A-11073, RRID: AB_2534117), AF488 goat anti-rabbit (1:1,000, Cat# A-11034, RRID: AB_2576217), AF488 goat anti-chicken (1:1,000, Cat# A-11039, RRID: AB_2534096), all from Life Technologies (Carlsbad, CA, United States). We routinely performed negative controls without addition of primary antibodies to validate the specificity of the immunostainings.

### Fluorescence Imaging and Data Analysis

Wide-field fluorescence imaging was performed using an inverted motorized Axiovert 200 M microscope (Zeiss) as described previously (Nieweg et al., 2015; van Stegen et al., 2017) to image the cultured astrocytes that exhibited a very flat morphology on the plane cover slips. In brief, fluorescence images were obtained with a 12 bit CoolSnap ES2 CCD camera (Photometrics) using VisiView software (Visitron Systems). The following filter sets (Zeiss) were used: (1) excitation 485/20 nm, beam splitter 510 nm, emission 515/565 nm (GFAP; MAP2; EGFP); (2) excitation 545/25 nm, beam splitter 570 nm, emission 605/70 nm (Tau; vGlut1; NLG1; GluA1; GluA2). For data analysis, images were thresholded using ImageJ software. vGlut1 puncta located on cortical or cerebellar astrocytes were identified by analyzing overlay images (Supplementary Figure 2).

### Electrophysiology and Data Analysis

Whole-cell voltage-clamp recordings were obtained from the target astrocytes or neurons at room temperature using an Axopatch 200B patch-clamp amplifier and pClamp 11.0.3 software (Molecular Devices, SanJose, CA) as described previously (Mohrmann et al., 1999). The patch pipette was filled with intracellular solution containing (in mM): 110 KCl, 20 HEPES, 10 EGTA, 0.25 CaCl2 with pH adjusted to 7.3. The standard extracellular solution contained (in mM) 130 NaCl, 3 KCl, 2.5 CaCl_2_, 1 MgCl_2_, and 20 HEPES, with pH adjusted to 7.3. The high [K^+^] extracellular solution contained (in mM) 112 NaCl, 40 KCl, 2.5 CaCl_2_, 1 MgCl_2_, and 20 HEPES, with pH adjusted to 7.3. AMPA receptor-mediated miniature EPSCs (1 μM TTX, 10μM gabazine added) were recorded at a holding potential of -60 mV.

### Statistics

All data are given as means ± SEM. Statistical significance was determined by Student’s *t*-test, if applicable, or by one-way ANOVA in combination with Tuckey’s *post-hoc* test by using SigmaPlot 11 software.

## Results

### Cortical Neuron Axons Physically Contact Immature Astrocytes in Culture

To study synaptogenic events occurring between presynaptic axons and astrocytes, we used a co-culture system consisting of mouse cortical explants (embryonic day 18–19) with axon outgrowth and added dissociated astrocytes (Figures 1A,B). Using dissociated neurons as postsynaptic cells, a similar co-culture system has previously been described to enable efficient functional synapse formation (Gottmann et al., 1997; Mohrmann et al., 1999; Figure 1C). Two types of immature astrocytes were used as potential target cells for cortical axons: GFAP-immunopositive astrocytes from newborn (P0) mouse cortex, and GFAP-immunopositive astrocytes from P7 mouse cerebellum. At 12 days in co-culture, cerebellar astrocytes showed a more ramified morphology (Figure 1E) as compared to the processes-lacking morphology of cortical astrocytes (Figure 1D), suggesting that cortical astrocytes were more immature. As positive control for synapse formation, dissociated MAP2-immunopositive neurons from embryonic day 18–19 mouse cortex were used as target cells (Figure 1F). To study whether cortical axons physically contacted immature astrocytes in a way comparable to contacting cortical neurons, we immunostained axons for tau protein, and imaged axon-astrocyte contacts (Figures 1G,H). We observed that cortical axons physically contacted both, cortical and cerebellar astrocytes in a way qualitatively comparable to contacting neurons (Figure 1I). This indicates that the basic physical contacts required for synapse formation between axons and immature astrocytes were established in culture.

### Immature Cortical Astrocytes Induce Synaptic Vesicle Clustering in Contacting Axons

**FIGURE 2:**
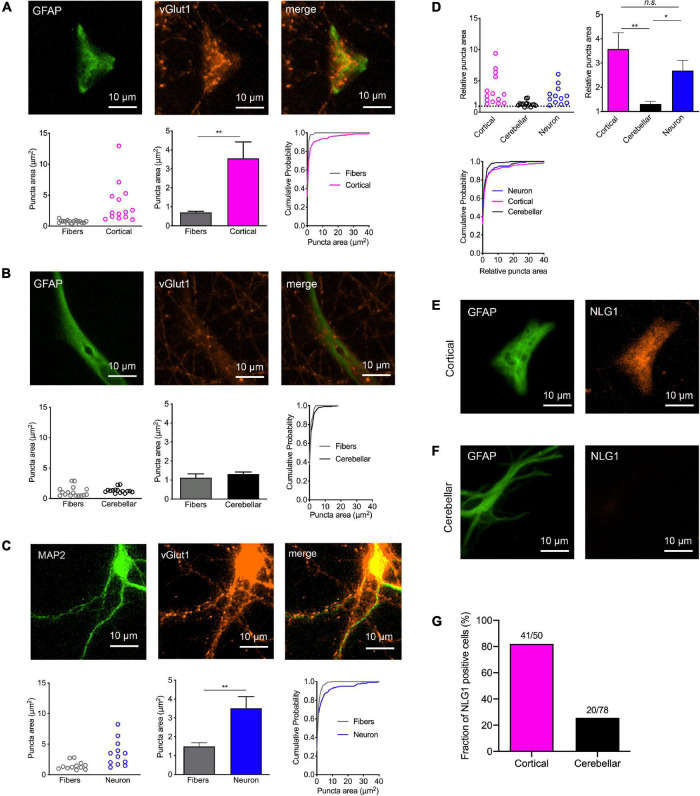
Induction of synaptic vesicle clusters in axons by immature cortical astrocytes. **(A–D)** Immunostaining for vGlut1 revealed induction of vesicle clusters in axons by target cells. **(A)** Upper panel: Immunostaining of immature cortical astrocytes for GFAP (left image), vGlut1 (middle image), and merge image. Lower panel: Quantification of vesicle cluster area [left: means of individual cells (*n* = 14/14); middle: means of cells ± SEM; right: cumulative distribution of the areas of all puncta (110/326)]. **(B)** Upper panel: Immunostaining of cerebellar astrocytes for GFAP (left image), vGlut1 (middle image), and merge image. Lower panel: Quantification of vesicle cluster (vGlut1 puncta) area [left: means of individual cells (*n* = 15/15); middle: means of cells ± SEM; right: cumulative distribution of the areas of all puncta (66/211)]. **(C)** Upper panel: Immunostaining of control neurons for MAP2 (left image), vGlut1 (middle image), and merge image. Lower panel: Quantification of vesicle cluster area [left: means of individual cells (*n* = 12/12); middle: means of cells ± SEM; right: cumulative distribution of the areas of all puncta (224/124)]. **P < 0.01, Student′s *t*-test. **(D)** Direct quantitative comparison of relative vGlut1 puncta area [normalized to respective mean puncta area in non-contacting axons (fibers)] in immature cortical astrocytes, in cerebellar astrocytes, and in control neurons. Upper left: means of individual cells; upper right: means of cells ± SEM; lower left: cumulative distribution of the areas of all puncta. *P < 0.05, **P < 0.01, one-way-ANOVA with Tuckey’s *post-hoc* test. **(E–G)** Immunostainings for GFAP (left image) and Neuroligin1 (NLG1) in immature cortical astrocytes **(E)** and in cerebellar astrocytes **(F)** as target cells. Note the prominent expression of Neuroligin1 in immature cortical astrocytes. **(G)** Quantification of the fraction of Neuroligin1 expressing cortical and cerebellar astrocytes.

We next wanted to study, whether physical contact between axons and immature astrocytes is accompanied by clustering of synaptic vesicles in contacting axons similar to synaptogenesis in neurons. We visualized synaptic vesicle clusters in axons by immunostaining for the synaptic vesicle protein vGlut1. We analyzed the resulting fluorescent puncta in explant axons with and without contact to target cells by determining vGlut1 puncta area as a quantitative measure of vesicle clustering (Supplementary Figure 2). Unexpectedly, vGlut1 puncta in axons on immature cortical astrocytes (co-stained for GFAP) were significantly larger as compared to vGlut1 puncta in axons not contacting target cells (Figure 2A). Using control neurons as target cells (co-stained for MAP2), we observed a similar significant difference between vGlut1 puncta in axons on neurons and vGlut1 puncta in non-contacting axons (Figure 2C). In contrast, vGlut1 puncta in axons on cerebrellar astrocytes were not significantly different as compared to vGlut1 puncta in non-contacting axons (Figure 2B). We further directly compared puncta areas in axons on cortical astrocytes, cerebellar astrocytes, and control neurons after normalization to the mean puncta area in non-contacting axons. Mean puncta areas in both, axons on cortical astrocytes and axons on control neurons were significantly larger as compared to the mean puncta area in axons on cerebellar astrocytes (Figure 2D). There was no significant difference between mean puncta areas in axons on cortical astrocytes and in axons on neurons. These results unexpectedly indicated that immature cortical astrocytes have a similar vesicle clustering effect on contacting axons as control cortical neurons.

Expression of the postsynaptic adhesion protein Neuroligin1 is well known to induce synaptic vesicle clustering in contacting axons (Scheiffele et al., 2000; Biederer et al., 2002; Dagar and Gottmann, 2019). Because expression of Neuroligins has recently been described in cortical astrocytes (Stogsdill et al., 2017), we qualitatively studied the expression of Neuroligin1 in the target astrocytes in our co-culture system. Immunostaining for Neuroligin1 revealed prominent expression of Neuroligin1 in immature cortical astrocytes (Figure 2E; 82% of GFAP-co-stained cells were immunopositive), whereas the vast majority of cerebellar astrocytes did not exhibit expression of Neuroligin1 (Figure 2F; only 26% of GFAP-co-stained cells were immunopositive). This result suggests that expression of Neuroligin1 selectively by immature cortical astrocytes might underlie the synaptogenic effect of cortical astrocytes on contacting axons.

### AMPA Receptor Subunit Expression in Innervated Immature Astrocytes

**FIGURE 3:**
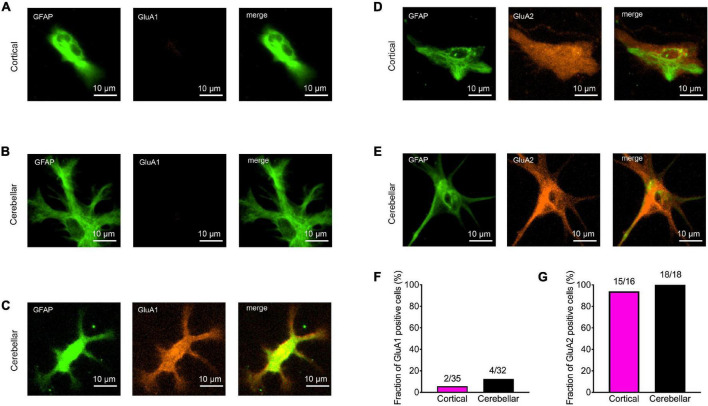
Expression of AMPA receptor subunits GluA1 and GluA2 in immature cortical and cerebellar astrocytes contacted by explant axons. **(A–C)** Co-immunostainings for GFAP (green) and GluA1 (red) in immature cortical astrocytes **(A)** and in cerebellar astrocytes **(B,C)**. Scale bars: 10 μm. **(D,E)** Co-immunostainings for GFAP (green) and GluA2 (red) in immature cortical astrocytes **(D)** and in cerebellar astrocytes **(E)**. **(F,G)** Quantification of GluA1 **(F)** and GluA2 **(G)** positive astrocytes contacted by explant axons.

We next wanted to study the expression of AMPA receptor subunits in immature cortical and cerebellar astrocytes that served as target cells. Therefore, we performed immunocytochemical co-stainings for GFAP and either GluA1 or GluA2 in astrocytes contacted by explant axons at 12 days in co-culture. In immature cortical astrocytes expression of GluA1 was almost completely absent (Figures 3A,F), while in cerebellar astrocytes a small fraction (around 10%) of cells expressed GluA1 (Figures 3B,C,F). In contrast to GluA1, GluA2 was expressed in almost all immature cortical (Figures 3D,G) and cerebellar astrocytes (Figures 3E,G) contacted by explant axons. This is in line with previous studies describing expression of GluA2 and GluA1 in cultured astrocytes (Backus et al., 1989; Condorelli et al., 1993).

### LRRTM2 Expression in Immature Astrocytes Induces Vesicle Clustering in Contacting Axons

**FIGURE 4:**
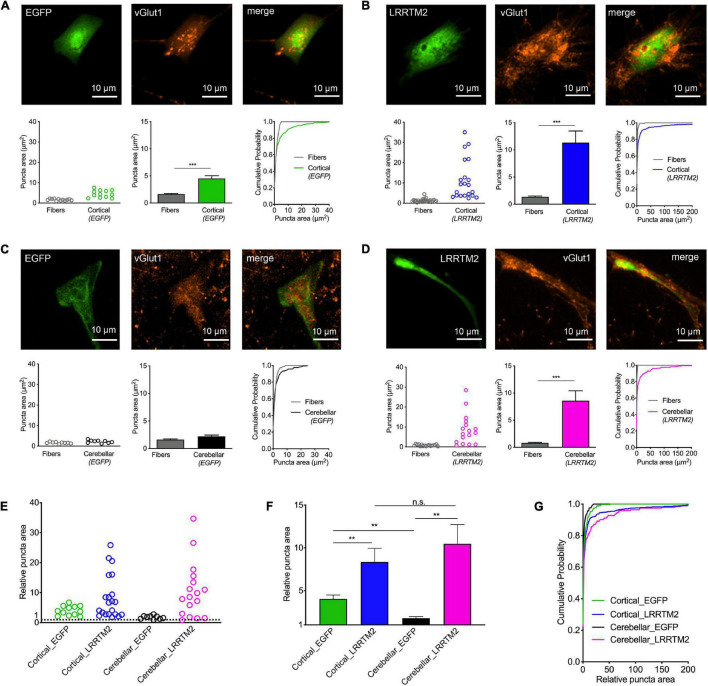
Induction of synaptic vesicle clusters in axons by LRRTM2 expression in immature cortical and cerebellar astrocytes. **(A–D)** Immunostaining for vGlut1 revealed induction of vesicle clusters in axons by LRRTM2 expression in target cells. **(A)** Expression of only EGFP (control) in immature cortical astrocytes. Upper panel: EGFP fluorescence (left image), immunocytochemistry for vGlut1 (middle image), and merge image. Lower panel: Quantification of vesicle cluster area on target cells (green) and in non-contacting axons (fibers) [left: means of individual cells (*n* = 12/12); middle: means of cells ± SEM; right: cumulative distribution of the areas of all puncta (86/300)]. **(B)** Expression of LRRTM2 + EGFP (co-transfection) in immature cortical astrocytes. Upper panel: EGFP fluorescence (left image), immunocytochemistry for vGlut1 (middle image), and merge image. Lower panel: Quantification of vesicle cluster area on target cells (blue) and in non-contacting axons (fibers) [left: means of individual cells (*n* = 21/21); middle: means of cells ± SEM; right: cumulative distribution of the areas of all puncta (177/462)]. **(C)** Expression of only EGFP (control) in cerebellar astrocytes. Upper panel: EGFP fluorescence (left image), immunocytochemistry for vGlut1 (middle image), and merge image. Lower panel: Quantification of vesicle cluster area on target cells (black) and in non-contacting axons (fibers) [left: means of individual cells (*n* = 9/9); middle: means of cells ± SEM; right: cumulative distribution of the areas of all puncta (72/163)]. **(D)** Expression of LRRTM2 + EGFP (co-transfection) in cerebellar astrocytes. Upper panel: EGFP fluorescence (left image), immunocytochemistry for vGlut1 (middle image), and merge image. Lower panel: Quantification of vesicle cluster area on target cells (magenta) and in non-contacting axons (fibers) [left: means of individual cells (*n* = 17/17); middle: means of cells ± SEM; right: cumulative distribution of the areas of all puncta (113/194)]. **(E–G)** Quantitative comparison of the effect sizes of EGFP (control) and LRRTM2 + EGFP expression in immature cortical and cerebellar astrocytes (calculated from data in **A–D**). vGlut1 puncta areas on target cells were normalized to the respective mean puncta area in non-contacting axons. **(E)** Means of normalized puncta areas of individual cells. **(F)** Means of cells ± SEM. **(G)** Cumulative distributions of the normalized areas of all puncta. ***P < 0.001; Student′s *t*-test **(A–D)**; **P < 0.01; one-way ANOVA with Tuckey’s *post-hoc* test **(F)**.

In a further approach intended to induce synaptogenic events, we used overexpression of the synaptogenic adhesion protein LRRTM2 (de Wit et al., 2009; Ko et al., 2009; Linhoff et al., 2009) in immature cortical and cerebellar astrocytes that were contacted by cortical explant axons. Sparse transfection of individual astrocytes with LRRTM2 was done at 10 days of explant co-culture using plasmid carrying magnetic beads (van Stegen et al., 2017). To identify transfected neurons EGFP was co-expressed. 2 days after transfection, immunostaining for vGlut1 was performed and vGlut1 puncta on astrocytes were quantitatively analyzed (Figure 4). As expected from the results shown above in Figure 2, control immature cortical astrocytes (expressing only EGFP) exhibited a significantly increased area of vGlut1 puncta as compared to the area of vGlut1 puncta on axons not contacting target cells (Figure 4A), indicating a retrograde induction of synaptic vesicle clustering. Immature cortical astrocytes overexpressing LRRTM2 also showed a significantly increased area of vGlut1 puncta as compared to vGlut1 puncta on axons not contacting target cells. Interestingly, the increase in puncta area appeared to be much stronger in cortical astrocytes overexpressing LRRTM2 (Figure 4B). Control cerebellar astrocytes (expressing only EGFP) did not exhibit a significantly different area of vGlut1 puncta as compared to the area of vGlut1 puncta on non-contacting axons (Figure 4C). In contrast, cerebellar astrocytes overexpressing LRRTM2 showed a significantly increased area of vGlut1 puncta as compared to the area of vGlut1 puncta on non-contacting axons (Figure 4D), thus indicating an induction of synaptic vesicle clustering by LRRTM2 expression. To statistically compare effect sizes of LRRTM2 expression, we normalized mean vGlut1 puncta areas on different astrocytes to the respective mean area of vGlut1 puncta on axons not contacting target cells (Figures 4E–G). We observed a significantly increased mean relative vGlut1 puncta area in both immature cortical and cerebellar astrocytes expressing LRRTM2, when compared to their respective control astrocytes (expressing only EGFP). In summary, our results demonstrate that overexpression of LRRTM2 in immature cortical astrocytes led to an enhancement of the vesicle clustering activity of untransfected astrocytes. In cerebellar astrocytes, which lacked a basal vesicle clustering activity, overexpression of LRRTM2 led to an appearance of synaptogenic activity.

### AMPA Receptor Subunit Expression in Innervated, LRRTM2 Expressing Astrocytes

**FIGURE 5:**
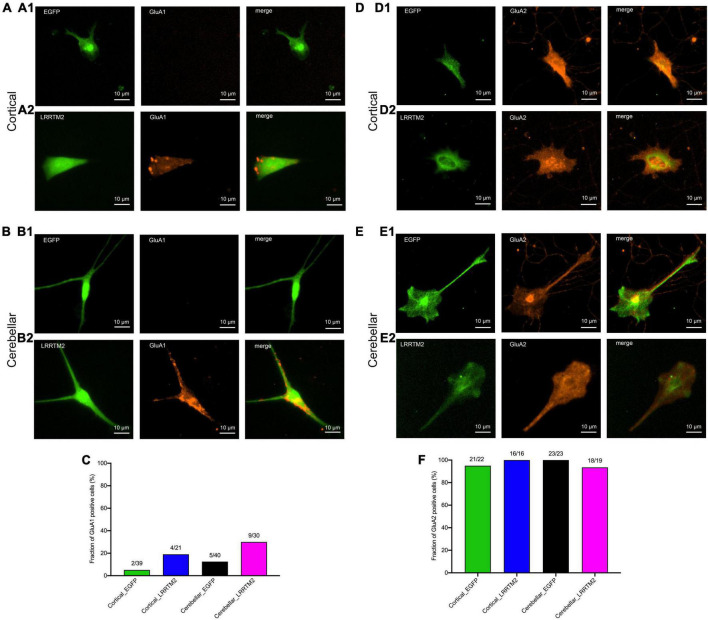
Effects of LRRTM2 expression on AMPA receptor subunit GluA1 and GluA2 expression in immature cortical and cerebellar astrocytes contacted by explant axons. **(A,B)** Fluorescence images of transfected astrocytes (EGFP (control) and LRRTM2 + EGFP, green) and immunostainings for GluA1 (red) in immature cortical astrocytes **(A1,A2)** and in cerebellar astrocytes **(B1,B2)**. Scale bars: 10 μm. **(C)** Quantification of the fraction of GluA1 positive astrocytes. **(D,E)** Fluorescence images of transfected astrocytes (EGFP (control) and LRRTM2 + EGFP, green) and immunostainings for GluA2 (red) in immature cortical astrocytes **(D1,D2)** and in cerebellar astrocytes **(E1,E2)**. **(F)** Quantification of the fraction of GluA2 positive astrocytes.

After having established the formation of presynaptic vesicle clusters on astrocytes by LRRTM2 expression, we wanted to study whether LRRTM2 expression in addition leads to an enhanced expression of AMPA receptor subunits. This might be expected, because LRRTM2 deficient neurons have been described to exhibit reduced AMPA receptor function and plasticity (Soler-Llavina et al., 2013; Bhouri et al., 2018). To qualitatively analyze AMPA receptor subunit expression, we performed immunocytochemistry for GluA1 and GluA2 in LRRTM2 (and EGFP) transfected, immature cortical and cerebellar astrocytes that were innervated by explant axons (12 days in co-culture). We observed a slight increase in the fraction of target cells immunopositive for GluA1 in both immature cortical and cerebellar astrocytes upon expression of LRRTM2 (Figures 5A2,B2,C). This parallel increase in GluA1 expressing cells might indicate an enhancing effect of LRRTM2 on AMPA receptor expression in astrocytes. However, the majority of transfected target astrocytes did not exhibit detectable GluA1 expression (Figures 5A1,B1,C). Qualitative immunocytochemical analysis for GluA2 subunit expression revealed that almost all transfected target astrocytes expressed GluA2 independent of LRRTM2 expression (Figures 5D–F).

### Lack of Miniature Excitatory Postsynaptic Currents in Innervated, LRRTM2 Expressing Astrocytes

**FIGURE 6:**
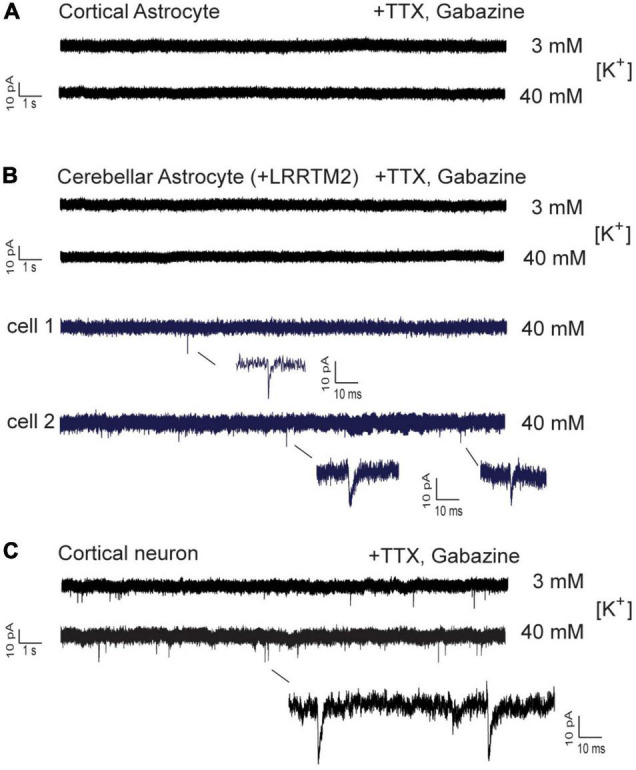
Whole-cell patch-clamp recordings in immature cortical astrocytes and in cerebellar astrocytes expressing LRRTM2. AMPA receptor-mediated miniature EPSCs (AMPA mEPSCs) were pharmacologically isolated by addition of TTX (1 μM) and gabazine (10 μM). Holding potential: -60 mV. Extracellular solution contained standard (3 mM) or elevated (40 mM) [K^+^] as indicated. **(A)** Lack of spontaneous AMPA mEPSCs in immature cortical astrocytes contacted by explant axons. *n* = 16 cells. **(B)** Lack of spontaneous AMPA mEPSCs in cerebellar astrocytes expressing LRRTM2 and contacted by explant axons. In a total of 16 cells only three individual mEPSC-like events were detected and are shown enlarged (blue). **(C)** Typical AMPA mEPSCs were routinely recorded in cortical neurons innervated by explant axons. All cells recorded at 12 days in explant co-culture.

We next wanted to study, whether AMPA receptor mediated postsynaptic responses can be observed in astrocytes innervated by explant axons. We attempted to record spontaneous AMPA receptor-mediated miniature excitatory postsynaptic currents (AMPA mEPSCs) from axon-contacted astrocytes by whole-cell patch-clamp recording. We used recording conditions with i) standard (3 mM) extracellular [K^+^] and with ii) elevated (40 mM) extracellular [K^+^] to stimulate vesicle release. In control cortical neurons innervated by explant axons at 12 days of co-culture typical spontaneous AMPA mEPSCS were present at both conditions in all 18 neurons recorded (Figure 6C). In immature cortical astrocytes (*n* = 16 cells) no AMPA mEPSCs were detectable (Figure 6A) despite the presence of presynaptic vesicle clusters and the expression of GluA2 subunits. Similar, in cerebellar astrocytes expressing LRRTM2 (*n* = 16 cells) AMPA mEPSCs were not detectable except for three individual mEPSC-like events (Figure 6B). Again, AMPA mEPSCs were largely absent despite the presence of presynaptic vesicle clusters and the expression of both GluA1 and GluA2 in about 1/3 of cerebellar astrocytes. In summary, expression of a single type of synaptogenic protein, LRRTM2, was not sufficient to reliably induce functional synapses characterized by postsynaptic currents in astrocytes.

## Discussion

In this paper, we analyzed as a first step the synaptogenic effects of co-cultured cortical and cerebellar astrocytes on axons growing out from cortical explants. Unexpectedly, we found that immature cortical astrocytes strongly increased presynaptic vesicle clustering in contacting cortical axons. By contrast, cerebellar astrocytes did not induce a comparable hemisynapse formation. Interestingly, the ability to induce hemisynapse formation correlated with the expression of Neuroligin1 in immature cortical astrocytes. Because Neuroligin1 is a well-established postsynaptic adhesion molecule with strong synaptogenic properties (Scheiffele et al., 2000; Biederer et al., 2002; Dagar and Gottmann, 2019), the expression of Neuroligin1 might underly the observed synaptogenic effect of immature cortical astrocytes. Expression of Neuroligins in cortical astrocytes in culture and *in vivo* has been well described (Zhang et al., 2014; Stogsdill et al., 2017), and has been shown to play an important role in astrocyte morphogenesis and neuronal synaptogenesis (Stogsdill et al., 2017).

In a second step, we expressed the synaptogenic protein LRRTM2 in immature cortical and in cerebellar astrocytes contacted by co-cultured explant axons. LRRTM2 has been shown to exhibit strong synaptogenic activity as indicated by presynaptic vesicle clustering (de Wit et al., 2009; Ko et al., 2009; Linhoff et al., 2009; Dagar and Gottmann, 2019), and to bind to presynaptic neurexins thereby inducing hemisynapse formation on the presynaptic side (de Wit et al., 2009; Ko et al., 2009; Siddiqui et al., 2010). As expected, we found an enhanced hemisynapse formation in immature cortical astrocytes upon expression of LRRTM2. Importantly, we further observed a strong induction of vesicle clustering in axons contacting cerebellar astrocytes, which did not induce hemisynapse formation without expression of LRRTM2.

To investigate functional synapse formation between explant axons and co-cultured astrocytes, we used whole-cell patch-clamp recording of AMPA receptor mediated miniature EPSCs. Recording of mEPSCs represents a highly sensitive method to detect spontaneous vesicle release, if functional AMPA receptors are expressed spatially aligned on the postsynaptic side. In GluR2 (GluA2) subunit overexpressing HEK293 cells contacted by presynaptic axons, spontaneous mEPSCs have been described upon expression of Neuroligin1 to induce presynaptic differentiation (Biederer et al., 2002; Fu et al., 2003; Sara et al., 2005). Unexpectedly, we did not observe spontaneous AMPA mEPSCs in immature cortical and in cerebellar astrocytes, although these cells strongly expressed GluA2 subunits and expressed synaptogenic proteins such as Neuroligin1 and LRRTM2 that induced presynaptic vesicle clustering. Only extremely few AMPA mEPSC-like events were found in very few astrocytes despite high [K^+^] induced depolarization. This lack of functional synapse formation between explant axons and co-cultured astrocytes might be due to presynaptic or postsynaptic reasons, as will be discussed below.

Presynaptically, the vesicle clusters induced by co-cultured astrocytes in contacting axons might not be capable of spontaneously releasing vesicles. However, this appears highly unlikely, because vesicle clusters induced by heterologous expression of Neuroligin1 (Biederer et al., 2002; Fu et al., 2003; Sara et al., 2005) and of LRRTM2 (de Wit et al., 2009; Linhoff et al., 2009) in HEK293 cells have been described to spontaneously release transmitter. Moreover, it is well known that even axonal vesicle clusters not in contact with a postsynaptic cell are capable of vesicle release (Matteoli et al., 1992; Kraszewski et al., 1995). Nevertheless, a lack of spontaneous vesicle release cannot be completely excluded, however, this potential explanation would require yet unknown factors from the co-cultured astrocytes that strongly inhibit spontaneous vesicle release.

Postsynaptically, we observed prominent expression of the AMPA receptor subunit GluA2 (GluR2) in co-cultured cortical and cerebellar astrocytes, and also expression of GluA1 (GluR1) in a fraction of LRRTM2 expressing astrocytes. Nevertheless, the co-cultured astrocytes might not express AMPA receptors on their plasma membrane resulting in a lack of postsynaptic currents. However, this appears highly unlikely, because functional surface AMPA receptors in cultured astrocytes have been well described in a large number of studies (e.g., Sontheimer et al., 1988; von Blankenfeld and Kettenmann, 1991; Burnashev et al., 1992; Gallo et al., 1994; David et al., 1996; Ceprian and Fulton, 2019). A hypothetical lack of surface AMPA receptors would require that the assembly machinery of AMPA receptors in the ER is not functional for example due to dysfunction of accessory proteins (e.g., ABHD6, TARPs and others; Schwenk and Fakler, 2021). Furthermore, in HEK293 cells expression of GluR2 (GluA2) subunits has been demonstrated to be sufficient to observe AMPA receptor mediated ion currents (Biederer et al., 2002; Sara et al., 2005). HEK293 cells most likely express AMPA receptor subunits encoded on plasmids at a high level, possibly resulting in the presence of functional AMPA receptors on the entire surface membrane. This might be the reason why miniature-like postsynaptic currents are readily observed in HEK293 cells. In contrast, in astrocytes AMPA receptor surface expression is likely to be more restricted by mechanisms regulating endogenous AMPA receptor expression.

Postsynaptic AMPA receptors have a relatively low affinity for binding glutamate as compared to NMDA receptors (for review see Traynelis et al., 2010). Therefore, a relatively high concentration of glutamate (mM) must be reached to activate AMPA receptor-mediated ion currents. In addition, AMPA receptors show strong desensitization thus requiring that such relatively high glutamate concentrations are reached within a millisecond (Lisman et al., 2007). Because miniature EPSCs are postsynaptic responses to the spontaneous release of just one synaptic vesicle, it appears to be critical that presynaptic vesicle release and postsynaptic AMPA receptors are spatially highly aligned to enable fast rising, high glutamate concentrations at the postsynaptic AMPA receptors (Lisman et al., 2007).

In line with this classic view, it has been found using modern super-resolution techniques that postsynaptic AMPA receptors are clustered in nanoscale subdomains (“slots”) that restrict their diffusional mobility in the plane of the postsynaptic membrane (for review see Opazo et al., 2012; Choquet and Hosy, 2020). Moreover, it has been described recently that molecularly defined presynaptic vesicle release sites are tightly aligned to these postsynaptic AMPA receptor nanoclusters thus forming a transsynaptic unit or nanocolumn (Tang et al., 2016; Biederer et al., 2017; Heine and Holcman, 2020). This delicate transsynaptic molecular architecture of functional synapses is presumably not established in the *in vitro* axon-astrocyte contacts studied in our present paper. The absence of transsynaptic nanocolumns might be the molecular reason, why we do not observe spontaneous AMPA mEPSCs despite the presence of presynaptic vesicle clusters and the expression of postsynaptic AMPA receptor subunits. In line with this idea, it has been demonstrated experimentally that interfering with the nanoscale spatial coupling of the presynapse-organizing protein Neuroligin1 and AMPA receptor nanodomains results in a significant decrease in AMPA mEPSC amplitudes (Haas et al., 2018).

We conclude that *in vitro* physical contacts of cortical neuron axons and target astrocytes enable the formation of hemisynapses, i.e., presynaptic differentiation in the contacting axons. This appears to require the expression of synaptogenic proteins such as Neuroligin1 and LRRTM2 by the astrocytes. However, AMPA mEPSCs, which are characteristic of fully functional synapses, were not observed despite the expression of AMPA receptor subunits in the target astrocytes. The lack of AMPA mEPSCs might indicate the absence of transsynaptic nanocolums tightly aligning vesicle release sites and AMPA receptor nanodomains at the molecular level.

## Data Availability Statement

The raw data supporting the conclusions of this article will be made available by the authors, without undue reservation.

## Ethics Statement

The animal study was reviewed and approved by the Tierschutzbeauftragter University Düsseldorf.

## Author Contributions

ZT performed the experiments and wrote the manuscript. KG designed the research and wrote the manuscript. Both authors contributed to the article and approved the submitted version.

## Conflict of Interest

The authors declare that the research was conducted in the absence of any commercial or financial relationships that could be construed as a potential conflict of interest.

## Publisher’s Note

All claims expressed in this article are solely those of the authors and do not necessarily represent those of their affiliated organizations, or those of the publisher, the editors and the reviewers. Any product that may be evaluated in this article, or claim that may be made by its manufacturer, is not guaranteed or endorsed by the publisher.

## Supplementary Material

Supplementary Figure 1 Validation of co-expression of myc-tagged LRRTM2 and EGFP in transfected astrocytes. Standard cultures of astrocytes without explants were co-transfected with EGFP and myc-LRRTM2 plasmids. **(A,C)** Co-expression of EGFP and myc-tagged LRRTM2 in cultured cortical **(A)** and cerebellar **(C)** astrocytes was confirmed by EGFP fluorescence (EGFP) and by immunocytochemical staining with myc-antibodies (myc). Overlay of images (merge) to demonstrate co-expression. **(B,D)** Control experiments, in which cultured cortical **(B)** and cerebellar **(D)** astrocytes were only transfected with EGFP, did not exhibit any myc fluorescence signal.

Supplementary Figure 2 Quantitative analysis of vGlut1-immunopositive puncta area. **(A,D)** Merged images of vGlut1 (red) and GFAP (green) immunofluorescence of an immature cortical astrocyte innervated by explant fibers **(A)** and of a cerebellar astrocyte innervated by explant fibers **(D)**. The astrocyte is outlined in red. The blue square demarcates the area in which vGlut1 puncta on explant fibers were analyzed at 12 days in co-culture. Scale bars: 10 μm. **(B,E)** Negative images of vGlut1 immunofluorescence that were thresholded to obtain the images shown in **(C,F)**. **(C,F)** Astrocyte outline (red) and explant fiber area (blue) were transferred onto thresholded negative images of vGlut1 immunofluorescence. Regions of interest (ROIs) were created around black puncta, and puncta area was determined separately for astrocytes (red outline) and explant fibers (blue square). ImageJ software was used for image analysis.
