# Supplementary figures and images for: Hemisynapse Formation Between Target Astrocytes and Cortical Neuron Axons in vitro
Source: Front Mol Neurosci. 2022 Mar 21;15:829506. doi: 10.3389/fnmol.2022.829506 (PMC8978633; doi:10.3389/fnmol.2022.829506)

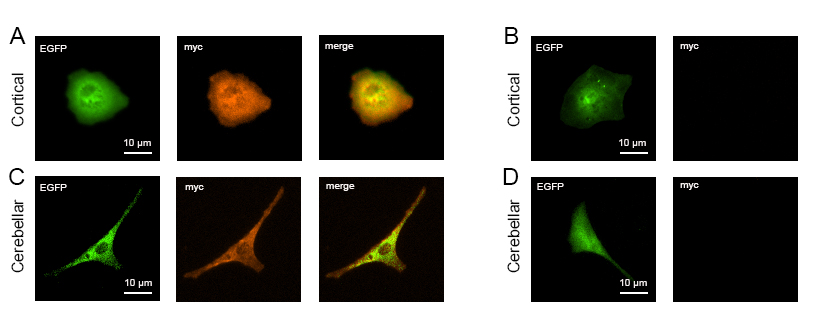

Supplement: Supplementary Figure 1 — Validation of co-expression of myc-tagged LRRTM2 and EGFP in transfected astrocytes. Standard cultures of astrocytes without explants were co-transfected with EGFP and myc-LRRTM2 plasmids. (A,C) Co-expression of EGFP and myc-tagged LRRTM2 in cultured cortical (A) and cerebellar (C) astrocytes was confirmed by EGFP fluorescence (EGFP) and by immunocytochemical staining with myc-antibodies (myc). Overlay of images (merge) to demonstrate co-expression. (B,D) Control experiments, in which cultured cortical (B) and cerebellar (D) astrocytes were only transfected with EGFP, did not exhibit any myc fluorescence signal. [file Image_1.JPEG]

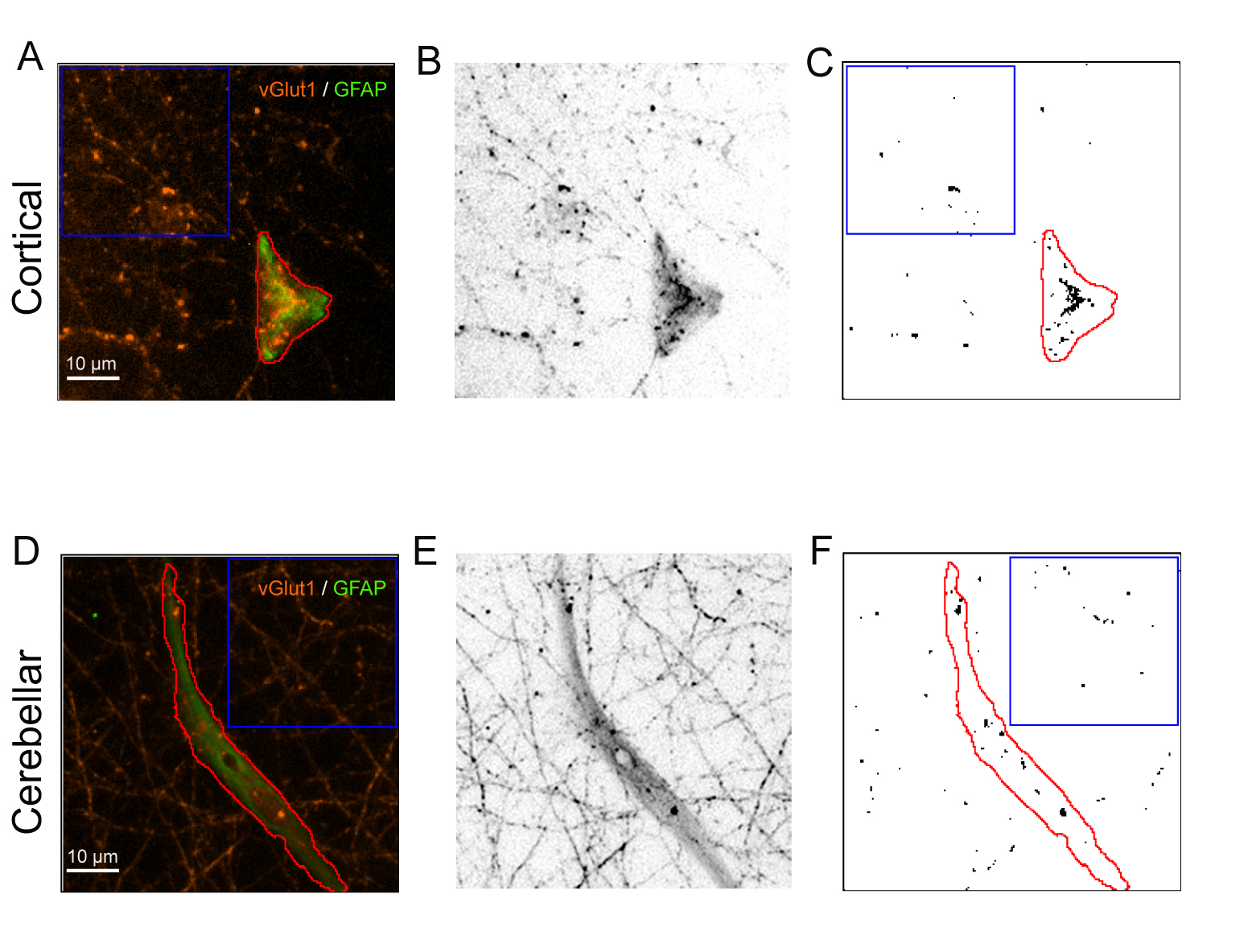

Supplement: Supplementary Figure 2 — Quantitative analysis of vGlut1-immunopositive puncta area. (A,D) Merged images of vGlut1 (red) and GFAP (green) immunofluorescence of an immature cortical astrocyte innervated by explant fibers (A) and of a cerebellar astrocyte innervated by explant fibers (D). The astrocyte is outlined in red. The blue square demarcates the area in which vGlut1 puncta on explant fibers were analyzed at 12 days in co-culture. Scale bars: 10 μm. (B,E) Negative images of vGlut1 immunofluorescence that were thresholded to obtain the images shown in (C,F). (C,F) Astrocyte outline (red) and explant fiber area (blue) were transferred onto thresholded negative images of vGlut1 immunofluorescence. Regions of interest (ROIs) were created around black puncta, and puncta area was determined separately for astrocytes (red outline) and explant fibers (blue square). ImageJ software was used for image analysis. [file Image_2.JPEG]
